# Red Blood Cell Transfusions and Iron Therapy for Patients Presenting with Acute Upper Gastrointestinal Bleeding: A Survey of Canadian Gastroenterologists and Hepatologists

**DOI:** 10.1155/2016/5610838

**Published:** 2016-06-28

**Authors:** Kyle J. Fortinsky, Myriam Martel, Roshan Razik, Gillian Spiegle, Zane R. Gallinger, Samir C. Grover, Katerina Pavenski, Adam V. Weizman, Lukasz Kwapisz, Sangeeta Mehta, Sarah Gray, Alan N. Barkun

**Affiliations:** ^1^Division of Gastroenterology, University of Toronto, Toronto, ON, Canada; ^2^Epidemiology and Biostatistics and Occupational Health, McGill University Health Center, McGill University, Montreal, QC, Canada; ^3^Division of Hematology and Transfusion Medicine, University of Toronto, Toronto, ON, Canada; ^4^Division of Gastroenterology, Western University, London, ON, Canada; ^5^Division of Critical Care, University of Toronto, Toronto, ON, Canada; ^6^Division of Emergency Medicine, University of Toronto, Toronto, ON, Canada; ^7^Division of Gastroenterology, McGill University Health Center, McGill University, Montreal, QC, Canada

## Abstract

*Introduction*. There is limited data evaluating physician transfusion practices in patients with acute upper gastrointestinal bleeding (UGIB).* Methods*. A web-based survey was sent to 500 gastroenterologists and hepatologists across Canada. The survey included clinical vignettes where physicians were asked to choose transfusion thresholds.* Results*. The response rate was 41% (*N* = 203). The reported hemoglobin (Hgb) transfusion trigger differed by up to 50 g/L. Transfusions were more liberal in hemodynamically unstable patients compared to stable patients (mean Hgb of 86.7 g/L versus 71.0 g/L; *p* < 0.001). Many clinicians (24%) reported transfusing a hemodynamically unstable patient at a Hgb threshold of 100 g/L and the majority (57%) are transfusing two units of RBCs as initial management. Patients with coronary artery disease (mean Hgb of 84.0 g/L versus 71.0 g/L; *p* < 0.01) or cirrhosis (mean Hgb of 74.4 g/L versus 71.0 g/L; *p* < 0.01) were transfused more liberally than healthy patients. Fewer than 15% would prescribe iron to patients with UGIB who are anemic upon discharge.* Conclusions*. The transfusion practices of gastroenterologists in the management of UGIB vary widely and more high-quality evidence is needed to help assess the efficacy and safety of selected transfusion thresholds in varying patients presenting with UGIB.

## 1. Introduction

The annual incidence of acute upper gastrointestinal bleeding (UGIB) in the United States has been reported as high as 160 per 100,000 adults leading to roughly 300,000 hospital admissions per year [1, 2]. Approximately 35–40% of patients presenting to hospital with UGIB are provided at least one red blood cell (RBC) transfusion [3]. In the UK, patients presenting with UGIB are transfused, on average, 1.58 units of RBCs [4]. UGIB accounts for 14% of all RBC transfusions in the UK [5]. Although the costs and complications of transfusions are well documented, clinicians often feel uncomfortable restricting transfusions, especially in patients with active hemorrhage and in those with significant comorbidities such as cardiac disease [6, 7].

Several randomized controlled trials (RCT) have investigated different transfusion strategies in critically ill patients and patients after cardiac and noncardiac surgeries [8–10]. The ideal transfusion threshold in patients presenting with UGIB remains largely unknown and has been studied in only one RCT which found that a more restrictive transfusion strategy may have mortality benefit in certain patients [11].

The 2013 American College of Gastroenterology (ACG) guidelines as well as international consensus guidelines suggest that patients with hemoglobin levels ≤70 g/L should receive blood transfusions to reach a target hemoglobin level of 70–90 g/L, provided that the individual has no coronary artery disease, evidence of tissue hypoperfusion, or acute hemorrhage [2, 12]. In patients with acute coronary syndrome, UGIB is associated with a markedly increased mortality, and a higher hemoglobin target level may be advantageous [2, 13].

In a 2011 UK audit, 43% of patients with UGIB received a RBC transfusion despite 73% presenting with hemoglobin above 80 g/L [7]. This audit, however, was performed prior to Villanueva et al.'s study that highlighted the benefits of restrictive transfusions [11].

Current international consensus and ACG guidelines on UGIB do not discuss the role of iron replacement therapy [2, 12]. Only one single centre RCT has evaluated iron replacement in patients after UGIB and found that patients who received iron therapy after UGIB had significantly lower rates of anemia at 3 months compared to placebo (17% versus 70%, *p* < 0.01) [14]. Correcting anemia after UGIB may minimize the need for transfusion during a rebleeding episode. One prospective observational study suggested that patients discharged after UGIB with hemoglobin values <100 g/L had twice the mortality rates when compared to patients with hemoglobin levels >100 g/L [15]. One retrospective study found that only 16% of anemic patients after UGIB are being prescribed oral iron supplementation upon discharge [16].

High-quality evidence guiding transfusion thresholds and iron therapy in the setting of UGIB is lacking. Many clinicians select transfusion thresholds based on individual patient factors and personal experience. The objectives of the current study were to characterize gastroenterologists' reported transfusion and iron prescribing practices in patients with UGIB. We also explored potential barriers for adopting results from large RCTs and guidelines into clinical practice.

## 2. Methods

### 2.1. Survey Participants

Responses included Canadian gastroenterologists, hepatologists, and current trainees of accredited Canadian gastroenterology and hepatology training programs. Email addresses of respondents were obtained by contacting institutions and by using publicly available information from institutional websites.

### 2.2. Survey Design

The survey was created using established methods to ensure optimal performance [17]. Demographics from the respondents were obtained to acquire baseline information regarding clinical expertise, comfort in managing UGIB, and sources of information used to base management approaches. A series of clinical vignettes were then presented with the purpose of determining transfusion thresholds ranging from 50 g/L to 120 g/L. Vignettes varied based upon patient age, comorbidities, presentation of GI bleeding (e.g., melena versus hematemesis), hemodynamic stability, and intravascular volume status (see survey scenarios as follows):

“Below what hemoglobin level would you transfuse red blood cells in this patient?”

 Scenario 1 (healthy, hemodynamically stable):
   A 50-year-old healthy woman presents with melena and is hemodynamically stable (BP 120/80, HR 65). There is no evidence of a volume deficit on clinical exam.
 Scenario 2 (healthy, hemodynamically unstable):
   A 50-year-old healthy woman presents with melena and is hemodynamically unstable (BP 90/50, HR 115) with evidence of a volume deficit on clinical exam.
 Scenario 3 (cardiovascular disease, hemodynamically stable):
   A 50-year-old man with triple vessel coronary artery disease presents with melena and is hemodynamically stable (BP 120/80, HR 65). There is no evidence of a volume deficit on clinical exam. The patient denies having any chest pain or dyspnea, and his ECG and troponin are unremarkable.
 Scenario 4 (cardiovascular disease, hemodynamically unstable):
   A 50-year-old man with triple vessel coronary artery disease presents with melena and is hemodynamically unstable (BP 90/50, HR 115) and he has ischemic ECG changes and an elevated troponin. He is complaining of mild chest pain and some shortness of breath.
 Scenario 5 (warfarin therapy, hemodynamically stable):
   A 65-year-old woman with hypertension and atrial fibrillation who is taking warfarin (INR 2.5) presents with melena and is hemodynamically stable (BP 120/80, HR 65). There is no evidence of a volume deficit on clinical exam.
 Scenario 6 (warfarin therapy, hemodynamically unstable):
   A 65-year-old woman with hypertension and atrial fibrillation who is taking warfarin (INR 2.5) presents with melena and is hemodynamically unstable (BP 90/60, HR 115). There is evidence of a volume deficit on clinical exam and the patient is being resuscitated with intravenous crystalloid.
 Scenario 7 (cirrhosis, hemodynamically stable):
   A 65-year-old patient with decompensated cirrhosis presents with hematemesis and is hemodynamically stable (BP 100/60, HR 85). There is no evidence of a volume deficit on clinical exam.

Additional multiple-choice questions assessed the number of units of red blood cells clinicians would transfuse as part of initial management. Further questions investigated the influence of warfarin intake compared to novel anticoagulants on respondents' transfusion thresholds and iron prescription rates for anemic patients after UGIB. We asked about physicians' awareness of current guidelines and potential barriers to adopting results of clinical trials and guidelines into clinical practice. The survey is available for viewing as a supplementary file.

### 2.3. Survey Distribution

Each potential participant was emailed two separate links to the survey, one in English and one in French. The questionnaire was disseminated using an online platform (https://www.surveymonkey.com/) in April 2015. Two follow-up emails were sent after the initial e-mail to encourage nonresponders at two-week intervals. Upon entering the survey, an introductory paragraph explained the purpose of the study. Participation was voluntary, and the respondent was asked to agree to participate and provide informed consent prior to initiating the survey. Participants were able to stop the survey at any time without penalty. All data was stored anonymously within the SurveyMonkey online database. The McGill University Research and Ethics Board approved the survey.

### 2.4. Statistical Analyses

Descriptive statistics including means, medians, and standard deviations for continuous variables and proportions with 95% confidence interval (95% CI) for categorical variables were used for each question in the survey. Categorical data were analyzed with *χ* ^2^ test or Fisher's exact test. Continuous data were analyzed with paired *t*-test. A preplanned regression model was performed using baseline characteristics of physicians including age, gender, level of training, years in practice, comfort managing bleeding, and knowledge of current guidelines to determine whether certain factors influenced transfusion practices, whereby *p* values < 0.05 were considered statistically significant. All statistical analyses were performed using SAS version 9.3 (SAS Institute, Cary, NC, USA).

## 3. Results

### 3.1. Baseline Characteristics

The survey was sent electronically to 518 gastroenterologists and hepatologists across Canada. Eighteen (3.5%) emails were undeliverable and therefore were removed from the database. Of the remaining 500 successfully sent emails, 204 participants entered the survey with 1 respondent not consenting to participate for an overall response rate of 41% (203/500). The majority of respondents (83.2%) were practicing staff physicians who, on average, manage approximately 63 cases of UGIB per year. Respondent characteristics are presented in Table 1. The sources of information drawn upon by participants to manage patients with UGIB are listed in Table 2.

### 3.2. Transfusion Thresholds Based upon Main Clinical Scenarios

**Figure 1:**
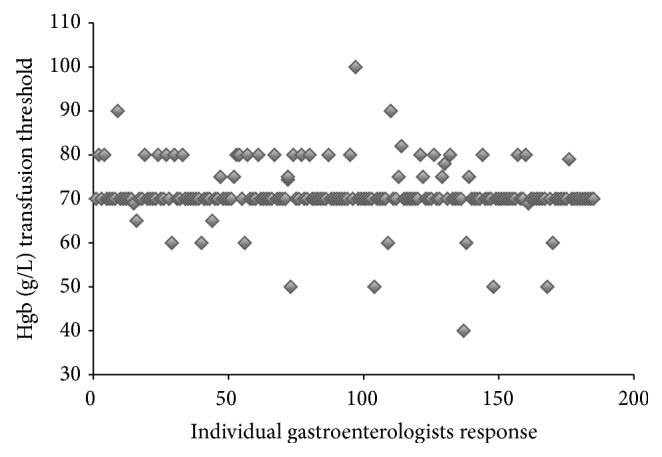
Selected hemoglobin transfusion thresholds for a healthy and hemodynamically stable patient with UGIB as described in Scenario  1. Each data point on the figure represents individual respondent's transfusion threshold.

**Figure 2:**
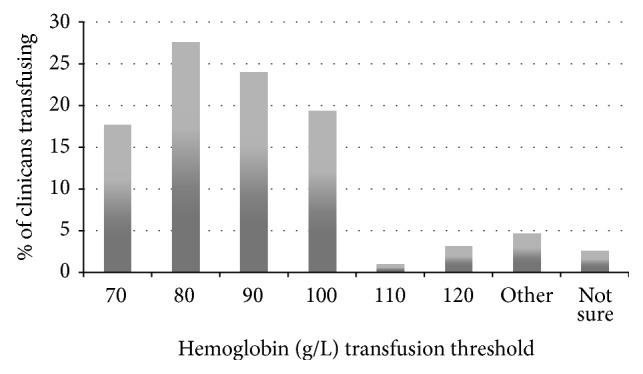
Selected hemoglobin transfusion thresholds for a healthy and hemodynamically unstable patient with UGIB as described in Scenario  2.

For Scenario 1 (hemodynamically stable 50-year-old healthy patient with melena), 71% of clinicians transfused at hemoglobin (Hgb) below 70 (mean: 71.0 ± 6.4) and 29% deviated from current guidelines (Figure 1). If the same patient was hemodynamically unstable (Scenario 2), 69.3% of clinicians would provide a transfusion at Hgb below 90 (mean: 86.7 ± 11.8). The distribution of transfusion thresholds for the hemodynamically unstable patient varied by up to 60 g/L (range: 60 g/L to 120 g/L; see Figure 2). Transfusion thresholds for the two scenarios differed significantly (71.0 versus 86.7 g/L, *p* < 0.001).

**Figure 3:**
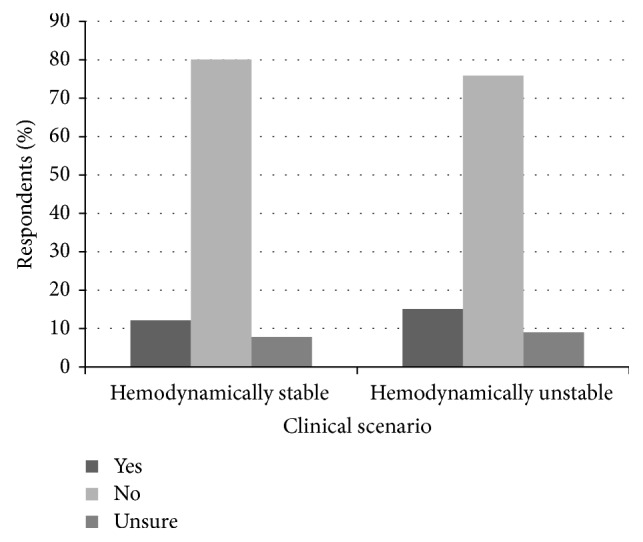
Are clinicians transfusing patients with UGIB on novel anticoagulants more liberally than patients on warfarin?

**Figure 4:**
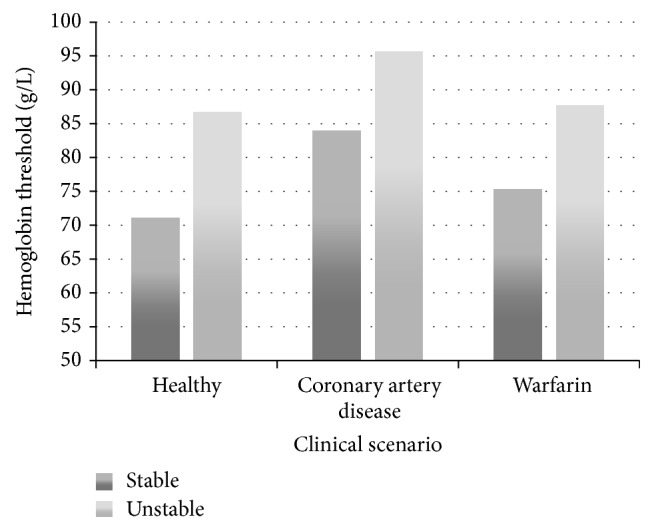
Mean hemoglobin transfusion threshold by clinical scenario.

Patients with coronary artery disease (mean Hgb 84.0 g/L versus 71.0 g/L, *p* < 0.001) or cirrhosis (mean Hgb 74.4 g/L versus 71.0 g/L, *p* < 0.01) were transfused at higher threshold hemoglobin than healthy patients, as were patients on warfarin (mean Hgb of 75.3 g/L versus 71.0 g/L, *p* < 0.001). 15% (95% CI) of respondents would perform transfusion more liberally if the patients were on dabigatran, rivaroxaban, or apixaban as opposed to warfarin (see Figure 3). Overall, hemodynamically unstable patients were transfused more liberally than hemodynamically stable patients across all scenarios (see Figure 4). For a healthy hemodynamically unstable patient (see survey scenarios in Section 2.2, Scenario  2) 19%, 1%, and 3% (95% CI) of clinicians would perform transfusion at hemoglobin threshold of 100 g/L, 110 g/L, and 120 g/L, respectively.

### 3.3. Initial Transfusion Management

Over half of respondents (57%) reported transfusing 2 units of RBCs as initial management. Most respondents (56.0% (48.4%; 63.4%)) also felt more likely to be held legally responsible for the complications related to “under-transfusing” than the complications associated with “over-transfusing.” In a clinical scenario targeting the most appropriate next step in management of an actively bleeding patient with UGIB, responses were highly varied with 38.6% (31.8–46.0%) awaiting an initial Hgb level to decide on a transfusion strategy (Table 3).

### 3.4. Iron Replacement Therapy

**Figure 5:**
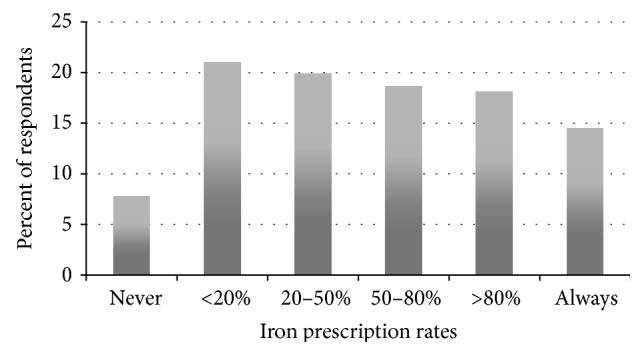
The percentage of clinicians who prescribe iron therapy to anemic patients after upper gastrointestinal bleeding.

Few gastroenterologists (14.5%, 9.9%; 20.6%) stated they routinely prescribe iron to patients with UGIB who are anemic at discharge (Figure 5). Most of these clinicians reported prescribing oral iron (81.3% (74.7%; 86.5%)) while very few are prescribing intravenous iron (5.4% (2.9; 10.0%)).

### 3.5. Adherence to Current Evidence and Guidelines

The majority of respondents were aware of the large RCT investigating transfusion thresholds in UGIB (92.2% (87.1%; 95.4%)) as well as existing international consensus guidelines endorsed and disseminated by the Canadian Association of Gastroenterology on UGIB (71.1% (63.8%; 77.4%)). However, roughly a quarter of these clinicians (22.9% (17.2%; 30.0%)) have not changed their transfusion practices based on either publication.  Table 4 shows potential barriers for adopting the results and recommendations from both the RCT and guidelines. Most gastroenterologists (60.8% (53.3%; 67.9%)) have never received formal education on transfusions in UGIB, and almost all (86.8% (80.8%; 91.1%)) feel an educational program or guideline would be useful.

### 3.6. Predictors of Transfusion Thresholds

The multivariate analysis did not reveal significant predictors of transfusion thresholds from possible preplanned candidate prognosticators.

## 4. Interpretation

There is wide variation in the transfusion thresholds amongst gastroenterologists and hepatologists managing patients with UGIB [7]. Even in a healthy and hemodynamically stable patient presenting with UGIB, a setting with the most explicit and agreed-upon guidelines, there were differences in transfusion thresholds of up to 60 g/L (threshold range from 40 g/L to 100 g/L) [2, 12]. Moreover, nearly 20% (95% CI) of clinicians perform transfusion more liberally than suggested by current guidelines (see Figure 1).

Respondents in the current study reported transfusing healthy and hemodynamically stable patients at a lower Hgb threshold than previous studies (71 g/L versus 77 g/L) [7]. Similar findings were noted in hemodynamically stable cirrhotic patients (74 g/L versus 85 g/L) [7]. The trend towards a more restrictive transfusion strategy may relate to the RCT by Villanueva et al. that showed a mortality benefit with restrictive transfusions, particularly in cirrhotic patients, possibly because blood transfusions may increase portal pressures and bleeding [11]. Indeed, 78% of respondents in our survey reported that their transfusion practices changed based on the results of this landmark trial in 2013.

The majority of clinicians (57%) report transfusing two units of RBCs as their initial management in a patient with UGIB. While one RBC unit can raise the Hgb by 10 g/L, one study found that this Hgb rise might be even greater in anemic patients [18, 19]. Only 10–15% of respondents were more liberal in transfusing a patient on a novel oral anticoagulant when compared to warfarin. One explanation for this finding may be the recent introduction of idarucizumab, an antidote for dabigatran [20].

Consistent with current guidelines, gastroenterologists seem to be more liberal in their transfusions when patients have underlying cardiac disease or present with signs of hemodynamic instability or volume depletion. One quarter (24%) of respondents reported transfusing a hemodynamically unstable patient at Hgb threshold of <100 g/L, while twice as many clinicians (50%) would perform transfusion at that same threshold if the patient also had cardiovascular disease.

The only three RCTs that have examined restrictive transfusions in patients with cardiovascular disease have excluded patients with gastrointestinal bleeding [8, 9, 21]. Villanueva et al.'s study excluded hemodynamically unstable patients and patients with underlying vascular disease. As such, evidence guiding transfusion management in these subsets of patients is lacking. Certain clinicians might believe that specific patients such as those with hemodynamic instability or cardiac disease may benefit from more liberal transfusions as such patients have not been accurately represented in the available literature.

Another possibility is that liberal transfusions may be a sign of “defensive medicine” whereby physicians act in a way to minimize legal liability [22]. In our study, 56% of respondents felt that they were more likely to be held legally responsible for the complications related to “under-transfusing” than the complications associated with “over-transfusing.” There are, however, dangerous consequences to overtransfusing patients including an increased risk of rebleeding and mortality as well as adverse transfusion reactions including anaphylactic reactions, transfusion-related circulatory overload, and transfusion-related acute lung injury [6, 8, 9, 11, 23–25].

The current study represents the fourth study to investigate the use of iron therapy in UGIB [14, 16, 26]. Less than 15% of respondents are consistently prescribing iron to anemic patients after UGIB, despite a recent RCT that found that iron therapy improves 28-day Hgb levels in anemic patients after UGIB [14].

A prospective, observational study would be the optimal environment for studying transfusion practices in UGIB. The previous UK survey found results that were slightly different compared to a national audit [7, 25, 27]. As such, it is possible that the data collected in our study does not accurately represent the true behavior of our respondents. An observational study, however, would not permit the examination of a large cohort of physicians across an entire country, which is what our survey accomplished. Furthermore, although a higher response rate is always preferable, it is unlikely that a larger sample size would have altered the results towards less heterogeneity in terms of transfusion practices. A previous study found that more recent graduates were more likely more restrictive in their transfusions which we were unable to confirm or refute [7]. It is possible that we were underpowered to detect this difference or that Villanueva et al.'s [11] study altered our respondents' practice.

Approximately 25% of Canadian gastroenterologists are not following current guidelines and are overtransfusing healthy and hemodynamically stable patients with UGIB. Over 97% of respondents are interested in an educational program or guideline to address transfusions in UGIB and 86% of respondents believe that such a program or guideline would change their management. Undoubtedly, the complexity of patient presentations with UGIB demands an individualized approach to transfusion management. Additional studies are needed to determine the effectiveness of iron therapy in UGIB and the safety of restrictive transfusion strategies in hemodynamically unstable patients and those with cardiac disease. More high-quality evidence will help to update guidelines to help reduce practice heterogeneity and improve patient outcomes [28].

## Figures and Tables

**Table 1:** Baseline characteristics of respondents.

Characteristics	Respondents, % (95% CI) (*N* = 203)
*Survey language*	
English	89.8% (84.9%; 93.2%)
French	10.2% (6.8%–15.2%)

*Age (years)*	
<36	32.2% (26.0%; 38.7%)
36–45	36.0% (29.7%; 42.8%)
46–55	14.3% (10.1%; 19.8%)
56–65	11.8% (8.1%; 17.0%)
>65	5.9% (3.5%; 11.2%)

*Female sex*	29.1% (23.3%; 35.7%)

*Province of practice*	
Western Canada (Alberta, British Columbia, Manitoba, and Saskatchewan)	27.8%
Ontario	49.8%
Quebec	16.8%
Atlantic Canada (New Brunswick, Newfoundland and Labrador, Nova Scotia, and Prince Edward Island)	4.6%
Outside of Canada	1%

*Level of training*	
Staff GI Physician	83.2% (77.5%; 87.8%)
GI trainee (PGY 4-5)	8.9% (5.7%; 13.6%)
GI trainee (PGY 6 or above) retired physician	6.4% (3.8%; 10.7%) 0.5% (0.0%; 2.8%)

*Type of center*	
Academic	41.9% (35.3%; 48.8%)
Community	27.1% (21.5%; 33.6%)
Combination of academic and community	13.8% (9.7%; 19.2%)
I am not affiliated with a hospital	0.5% (0.0%; 2.8%)

*Comfort managing UGIB*	
Extremely comfortable	66.3% (59.3%; 72.7%)
Slightly comfortable	24.6% (19.0%; 31.2%)
Extremely uncomfortable	5.4% (2.9%; 9.6%)
Neutral	3.7% (1.8%; 7.5%)

**Table 2:** Continuing medical education (CME) used for management of UGIB (*n* = 203).

Type of CME used (each respondent may select multiple choices)	
Medical conferences	81.5% (76.1%; 86.8%)
Clinical guidelines on UGIB management	69.8% (63.4%; 76.1%)
Review articles	65.4% (58.8%; 71.9%)
Primary journal articles	63.9% (57.3%; 70.5%)
Online clinical resources (e.g., up to date)	53.2% (46.3%; 60.1%)
Journal clubs	49.3% (42.4%; 56.2%)
Newsletters (e.g., NEJM journal watch)	30.7% (24.4%; 37.1%)
Online webinars or podcasts	7.8 (4.1%; 11.5%)
Other	2.4% (1.1%; 5.6%)
None	0.5% (0.0%; 1.4%)

**Table 3:** Clinicians deciding on the best next step in management of an actively bleeding patient with UGIB (see scenario below).

A 50-year-old healthy patient presents with hematemesis and is hemodynamically unstable (BP 90/50, HR 115) with evidence of a volume deficit on clinical exam. Two large bore IVs were inserted and resuscitation was initiated with intravenous crystalloid. Routine blood work including a CBC and RBC cross-match has been sent. What would be your next steps?	
I would hold off on a blood transfusion until I know the hemoglobin level	38.6% (31.8%; 46.0%)
I would wait for cross-matched RBCs and transfuse 1-2 units once available	25.0% (19.2%; 31.9%)
It depends on how much the patient appears to be bleeding	18.2% (13.2%; 24.5%)
I would transfuse 1-2 units of uncross-matched red blood cells STAT	13.1% (8.9%; 18.6%)
It depends on patient's symptoms	4.6% (2.3%; 8.7%)
I would wait for cross-matched RBCs and transfuse 3-4 units once available	0.6% (0.1%; 3.2%)

**Table 4:** Barriers to evidence-based practice.

*NEJM Study*: Why has this study NOT changed your transfusion practice? Choose as many as apply.	
My current practice was already in line with the conclusions of this study	59.4% (41.4%; 77.4%)
There can never be a “strict” transfusion cutoff; need a case-by-case basis	50.0% (31.7%; 68.3%)
The protocol in the study was not usual practice (i.e., endoscopy within 6 hours)	43.8% (25.6%; 61.9%)
More studies are required	28.1% (11.7%; 44.6%)
I wouldn't change my practice based on a single study	25.0% (9.1%; 40.9%)
The study was based out of a single center	12.5% (0.4%; 24.6%)
My patients are significantly different than those in the study	9.4% (0.0%; 20.1%)
I don't agree with the study analysis and/or conclusions	6.3% (0.0%; 15.1%)
Other	1.4% (0.4%; 4.9%)
I will never feel comfortable with restrictive transfusion	0.0%

*International Consensus Guidelines*	
Why do you NOT agree with these proposed transfusion thresholds? Choose as many as apply.	

Using strict cut-offs prevents using clinical judgement	73.7% (59.0%; 88.4%)
There is insufficient high quality evidence to support the cut-offs	36.8% (20.8%; 52.9%)
I was not aware of these cut-offs	7.9% (0.0%; 16.9%)
Patient outcomes are better with more liberal transfusion thresholds	5.3% (0.0%; 12.7%)
